# miR-27a-3p regulates the inhibitory influence of endothelin 3 on the tumorigenesis of papillary thyroid cancer cells

**DOI:** 10.3892/mmr.2021.12105

**Published:** 2021-04-19

**Authors:** Hongxin Chen, Binlin Cai, Kun Liu, Qingquan Hua

Mol Med Rep 23: 243, 2021; DOI: 10.3892/mmr.2021.11882

Following the publication of the above article, the authors have realized that Figs. 4 and 7 contained data panels that had been erroneously assembled in these figures. These errors arose as a consequence of miscommunication among several of the authors, and changes in personnel throughout the course of the project.

The revised versions of Figs. 4 and 7 are shown on the next page. Note that the replacement of the data shown also merits some changes being made to the descriptions in the Results section for the relevant figures (changed text is highlighted in bold). In the ‘*EDN3 overexpression in PTC cells impairs cell migration and invasion*’ subsection of the Results section in the right-hand column of p. 6, the second sentence should now read as follows: ‘To detect the invasiveness of TPC-1 and GLAG-66 cells, transwell assay was performed, and the results of the transwell assay indicated that the number of cells invaded decreased by about **40%** after transfecting with EDN3-OE (Fig. 4C and D)’. Secondly, the last sentence on p. 11 should be rephrased to the following: ‘The transwell assay results revealed that the invasion was enhanced in the miR-27a-3p mimic group and the invasion was suppressed in cells of the EDN3-OE group, compared with the control group and the co-transfection group (Fig. 7C and D)’.

All the authors approve of the publication of this corrigendum, and the authors are grateful to the Editor of *Molecular Medicine Reports* for granting them the opportunity to publish this. The authors regret that these errors were included in the paper, and also apologize to the readership for any inconvenience caused.

## Figures and Tables

**Figure 4.**
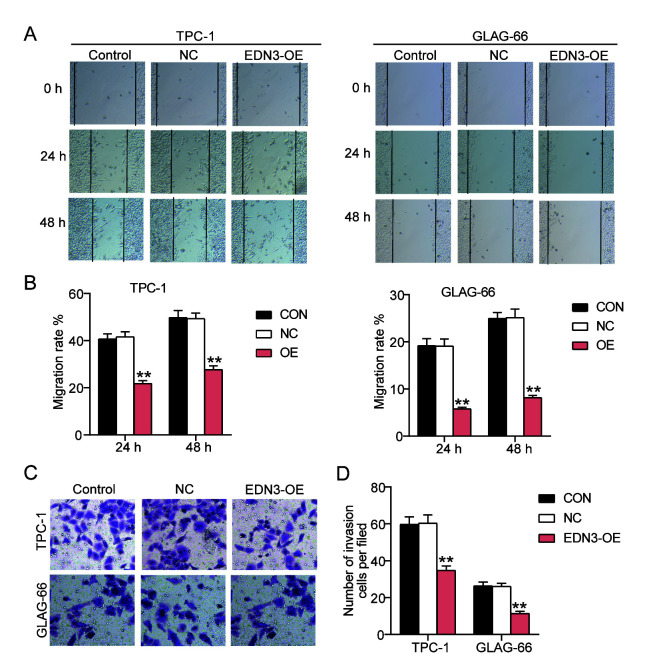
EDN3 overexpression inhibits the migration and invasion of PTC cells. (A and B) The ability of cell migration was assessed using wound healing assay following transfection of the TPC-1 and GLAG-66 cells for 24 and 48 h. Magnification, ×100. (C and D) Transwell assay was used to verify the invasion phenotype of TPC-1 and GLAG-66 cells following transfection with EDN3-OE plasmids or NC plasmids. Magnification, ×100. *P<0.05, **P<0.001 vs. the control group using one-way ANOVA. The bar represented the standard deviation of three independent experiments. EDN, endothelin; PTC, papillary thyroid cancer; EDN3-OE, TPC-1 and GLAG-66 cells transfected with EDN3-OE plasmids; NC, TPC-1 and GLAG-66 cells transfected with negative control plasmids; CON, TPC-1 and GLAG-66 cells were cultured without any treatments.

**Figure 7.**
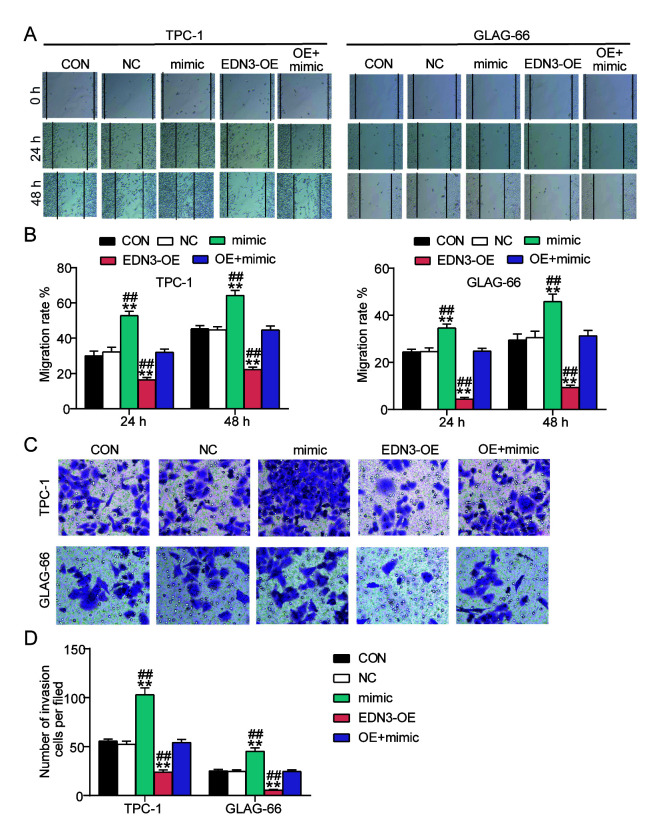
Overexpression of EDN3 reversed the promoting effects of miR-27a-3p on PTC cell migration and invasion. (A and B) Wound healing assay was conducted to measure the cell migration 24 and 48 h after the transfection. Magnification, ×100. (C and D) The number of invading cells was detected using transwell assay to reflect the invasion ability of TPC-1 and GLAG-66 cells. Magnification, ×100. *P<0.05, **P<0.001 vs. control group using one-way method. #P<0.05, ##P<0.001 vs. OE + mimic group using one-way ANOVA. The bar represented the standard deviation of at least three independent experiments. EDN, endothelin; PTC, papillary thyroid cancer; CON, TPC-1 and GLAG-66 cells were cultured without any treatments; NC, TPC-1 and GLAG-66 cells were transfected with negative control plasmids; EDN3-OE, TPC-1 and GLAG-66 cells were transfected with EDN3-OE plasmids; mimic, TPC-1 and GLAG-6 cells were transfected with miR-27a-3p mimic; OE + mimic, TPC-1 and GLAG-66 cells were co-transfected with EDN3-OE plasmids and miR-27a-3p mimic.

